# The Role of Probiotics in Enhancing Animal Health: Mechanisms, Benefits, and Applications in Livestock and Companion Animals

**DOI:** 10.3390/ani15202986

**Published:** 2025-10-15

**Authors:** Sorin Marian Mârza, Camelia Munteanu, Ionel Papuc, Lăcătuş Radu, Robert Cristian Purdoiu

**Affiliations:** 1 Clinical Sciences Department, Faculty of Veterinary Medicine, University of Agricultural Sciences and Veterinary Medicine, 3-5 Manastur Street, 400372 Cluj-Napoca, Romania; sorin.marza@usamvcluj.ro (S.M.M.); ionel.papuc@usamvcluj.ro (I.P.); radu.lacatus@usamvcluj.ro (L.R.); robert.purdoiu@usamvcluj.ro (R.C.P.); 2 Biology Section, Faculty of Agriculture, University of Agricultural Sciences and Veterinary Medicine, 3-5 Manastur Street, 400372 Cluj-Napoca, Romania

**Keywords:** probiotics, microorganism, livestock, gastrointestinal, microbiota, companion animal

## Abstract

**Simple Summary:**

This review highlights the diverse applications and benefits of probiotics in animal health, focusing on both livestock and companion animals. Probiotics enhance gut health, modulate the immune system, and suppress pathogens, leading to improved productivity and disease prevention. In livestock, they improve feed conversion ratios, mitigate methane emissions, and serve as natural alternatives to antibiotics, aligning with sustainability goals. In companion animals, probiotics alleviate gastrointestinal issues, reduce stress through the gut–brain axis, and address conditions like allergies.

**Abstract:**

This review examines the diverse ways in which probiotics, defined as live microorganisms that provide health benefits to the host when administered in adequate amounts, contribute to animal health and welfare across both livestock and companion species. By modulating gut microbiota, enhancing immune responses, and suppressing harmful pathogens, probiotics represent an effective strategy for disease prevention and performance improvement without reliance on antibiotics. In livestock production, these beneficial microbes have been shown to optimize feed utilization, support growth, and reduce methane emissions, thereby contributing to more sustainable farming practices. Their role extends beyond productivity, as probiotics also help mitigate antimicrobial resistance (AMR) by offering natural alternatives to conventional treatments. In aquaculture, they further promote environmental sustainability by improving water quality and reducing pathogen loads. For companion animals such as dogs and cats, probiotics are increasingly recognized for their ability to support gastrointestinal balance, alleviate stress through gut–brain axis interactions, and aid in the management of common conditions including diarrhea, food sensitivities, and allergies. The integration of probiotics into veterinary practice thus reflects a growing emphasis on holistic and preventive approaches to animal health. Despite these advances, several challenges remain, including variability in strain-specific efficacy, regulatory limitations, and cost-effectiveness in large-scale applications. Emerging research into precision probiotics, host–microbiome interactions, and innovative delivery methods offers promising avenues to overcome these barriers. As such, probiotics can be regarded not only as functional supplements but also as transformative tools that intersect animal health, productivity, and sustainability.

## 1. Introduction

**Figure 1:**
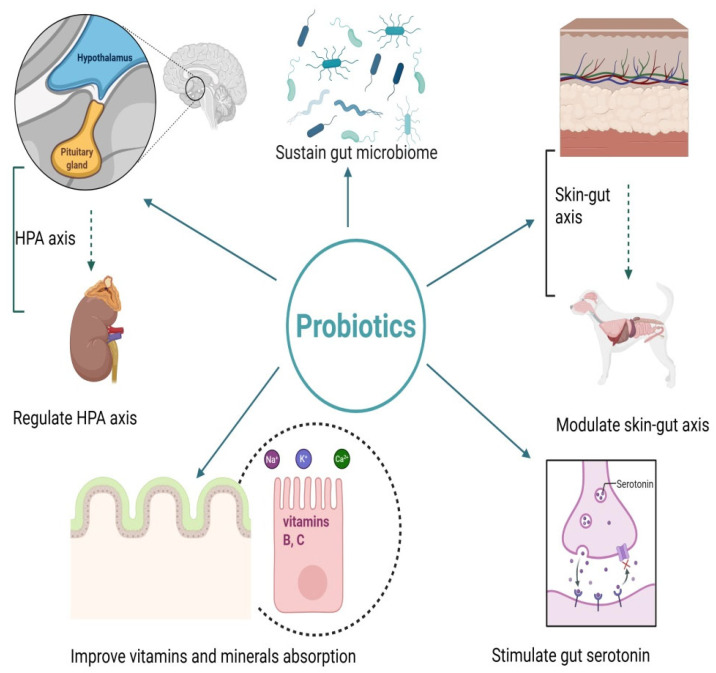
The Multifaceted Impact of Probiotics in Animals: Probiotics play a crucial role in maintaining an animal’s overall health in various ways. They maintain the intestinal flora, which is essential for a healthy internal environment. Another important mechanism for stress management that probiotics aid in regulating is the HPA axis. Through better absorption of vital minerals and vitamins, such as B, C, Na^+^, K^+^, and Ca^2+^, they improve overall nutrition. Additionally, probiotics contribute to the gut’s production of serotonin, a neurotransmitter that influences mood. Lastly, they alter the skin–gut axis, highlighting the close relationship between healthy skin and a functioning digestive tract.

The growing interest in sustainable and health-conscious practices in animal husbandry and pet care has brought probiotics to the forefront of veterinary research. Probiotics are live microorganisms that confer a health benefit to the host when administered in adequate amounts, and they have demonstrated potential to promote animal welfare, enhance immunity, and improve overall performance in livestock and companion animals [1,2]. Among the ones we can highlight, common probiotic taxa include *Lactobacillus*, *Bifidobacterium*, and *Enterococcus*, as well as the yeast *Saccharomyces boulardii*; these organisms interact with the gastrointestinal (GI) microbiota to optimize health outcomes [3]. In turn, one way to support these beneficial bacteria is through the use of prebiotics, which selectively stimulate resident microbes such as bifidobacteria and can offer more durable modulation of the gut microbiota than probiotics, whose effects may be transient [4]. In ruminants, probiotics can modulate rumen fermentation dynamics [5]. For example, probiotic supplementation has been shown to enhance rumen microbial crude protein synthesis and fiber digestibility while maintaining stable rumen fermentation and microbial ecology. Such findings illustrate how microbiome-aware probiotic interventions can align productivity with sustainability goals [6]. The mechanisms underlying probiotic effects are multifaceted, encompassing competitive exclusion of pathogens, production of antimicrobial compounds, reinforcement of epithelial barrier function, and modulation of host immune responses [7]. At the production level, probiotics have been associated with improved feed conversion ratios, reduced incidence of gastrointestinal disease, and enhanced growth performance, positioning them as economically viable complements or alternatives to traditional growth-promoting antibiotics [8]. Advances in microbiome research have clarified host–microbiota relationships and how probiotics can influence community composition and metabolism to bolster resilience against pathogenic challenges [9]. In poultry systems, where antibiotic growth promoters have been restricted or banned in many jurisdictions, probiotics are increasingly deployed as effective alternatives. Supplementation has been shown to strengthen gut integrity, lower pathogen loads, and improve nutrient utilization, thereby supporting more sustainable production [10,11]. Strains such as *L. plantarum* and *Propionibacterium freudenreichii* have been linked to reduced methane emissions and improved feed efficiency, underscoring a potential contribution to lowering the environmental footprint of ruminant agriculture [12]. In swine, probiotics represent a promising option for preventing and treating post-weaning diarrhea (PWD) by restoring intestinal microecological balance, strengthening mucosal and immune barriers, and promoting pathogen exclusion, antimicrobial production, toxin neutralization, and maintenance of barrier integrity [13]. In dogs, probiotics are used to manage lifestyle-associated perturbations of the microbiota and immune function. Clinical and translational evidence demonstrates improvements in gastrointestinal health, reductions in diarrhea frequency, and broader immunomodulatory effects, including attenuation of cutaneous inflammation in canine dermatitis. In cats, probiotics are likewise employed to support gastrointestinal health and to reduce the frequency of diarrhea. Emerging data suggest wider immunomodulatory benefits in felines, broadly comparable to those reported in dogs. Clinical and translational evidence indicates benefits for gastrointestinal health, reductions in diarrhea frequency, and broader immunomodulatory effects in dogs and cats [14,15]. Experimental work in stress-induced murine models further demonstrates psychobiotic effects. For example, oral administration of *L. plantarum* D-9 alleviated anxiety- and depression-like behaviors by modulating tryptophan metabolism, the hypothalamic–pituitary–adrenal axis, inflammation, and gut microbiota composition [16] (Figure 1). Despite the promising benefits of probiotics, challenges remain in their widespread adoption. These include variability in strain efficacy, lack of standardized dosing protocols, and gaps in understanding host-specific responses to different probiotics. For instance, in vivo studies in cattle have demonstrated inconsistent effects of probiotic strains on rumen fermentation and productivity [17]. In poultry, probiotic supplementation at different doses produced variable outcomes in growth and microbiota modulation [18]. Similar inconsistencies have been reported in piglets [19], while in companion animals, probiotics have shown mixed efficacy in managing diarrhea and other clinical conditions [20]. The economic benefits of probiotic use cannot be overlooked. By improving animal productivity and reducing the reliance on costly treatments for disease, probiotics present a cost-effective solution for farmers and pet owners alike [21]. For example, supplementation in dairy cattle has been linked to improved milk yield and lower veterinary expenditures [22], while in poultry, enhanced feed conversion efficiency translates into reduced production costs [10]. In swine, preventing post-weaning diarrhea through probiotic interventions can substantially decrease economic losses related to morbidity and medication [23]. In companion animals, probiotics have been reported to reduce the incidence of gastrointestinal disorders, lowering healthcare-related expenses for owners [24,25,26]. Moreover, public awareness regarding antibiotic resistance has created a growing demand for natural alternatives, further driving the adoption of probiotics in both commercial and domestic animal care [27].

Furthermore, regulatory frameworks governing probiotic use in animal feed vary widely across regions, complicating their commercialization and application. In the European Union, probiotics are classified as feed additives and require approval under Regulation (EC) No 1831/2003, with extensive safety and efficacy evaluations conducted by the European Food Safety Authority (EFSA). By contrast, in the United States, the Food and Drug Administration (FDA) generally regulates probiotics intended for animal use as feed ingredients or under the Generally Recognized as Safe (GRAS) system, which is less restrictive. In Asian countries, such as China and Japan, frameworks differ, with China implementing strict licensing procedures through the Ministry of Agriculture and Rural Affairs, while Japan has historically adopted a more permissive approach. These regulatory discrepancies create challenges for harmonization and for the global commercialization of probiotic products [28]. The use of probiotics in animal health has also been linked to the mitigation of chronic inflammatory conditions, as they can regulate cytokine production and balance pro-inflammatory and anti-inflammatory pathways [29]. For instance, strains such as *L. rhamnosus* have demonstrated the ability to enhance mucosal barrier function by increasing tight junction integrity in the gut epithelium [30]. This action is particularly relevant in preventing the translocation of pathogens, a common issue in intensive livestock farming environments [31]. These microorganisms are also capable of producing and degrading neuroactive compounds, thereby influencing host physiology through microbial endocrinology pathways. Recent work in poultry has shown that selected strains can both produce dopamine and degrade histamine in feed environments, providing proof-of-concept for designing microbial interventions with targeted neurochemical functions [32,33]. In aquaculture, probiotics are gaining traction as a sustainable method to improve water quality and reduce disease outbreaks, particularly in shrimp and fish farming [33]. Research shows that *B. subtilis* can outcompete harmful bacteria in aquatic systems, leading to healthier fish and improved production metrics [34]. Similarly, probiotics in aquaculture have been shown to enhance growth rates and feed efficiency while reducing the need for antibiotic treatments [35]. In aquaculture, pathogens such as *Vibrio harveyi* pose significant health and economic challenges. Beyond immune modulation, it is increasingly recognized that neurochemicals influence pathogen behavior; norepinephrine and dopamine have been shown to enhance *V. harveyi* motility, biofilm formation, and virulence [36]. These findings suggest that probiotic modulation of microbial endocrinology could represent a novel pathway for mitigating pathogenicity in aquaculture systems. Emerging fields such as precision microbiome engineering are poised to revolutionize probiotic applications by allowing targeted modulation of the gut microbiota for specific health outcomes [37]. Advances in sequencing technologies and bioinformatics are helping to identify novel probiotic strains with unique functional properties, such as antimicrobial peptide production and bile acid metabolism [38]. Insights from livestock and companion animals highlight the gut microbiota’s vital role in promoting resilience to environmental stressors—such as in hibernating species—and offer valuable perspectives for modulating the human microbiome to mitigate lifestyle disease complications [39]. The ability of probiotics to modulate the host’s gut–brain axis is a burgeoning area of research, particularly in companion animals, where stress-induced behaviors are a concern [40]. Specific strains such as *L. reuteri* have shown potential to reduce stress and improve social behaviors through the production of neuroactive compounds like gamma-aminobutyric acid (GABA) [41]. This connection between the gut microbiota and the central nervous system highlights probiotics’ broader implications beyond physical health [42]. In poultry production, the adoption of probiotics has been linked to significant reductions in Campylobacter and Salmonella colonization in the gut, addressing critical public health risks associated with zoonotic pathogens. The mechanism of action includes competitive exclusion and the secretion of bacteriocins that inhibit pathogenic bacteria [43]. Additionally, probiotics such as *P. acidilactici* have demonstrated resilience under harsh gastrointestinal conditions, making them effective candidates for large-scale use in poultry farming [44]. In swine, the administration of probiotics during gestation and lactation has been shown to improve sows’ gut health and positively influence the microbiota composition of their offspring [45]. Such maternal effects suggest that probiotics may play a role in early-life microbiome programming, with long-term benefits for animal health and performance [46]. The environmental implications of probiotics also merit attention. In aquaculture, the use of probiotics has been associated with a reduction in antibiotic residues and improved water quality, contributing to more sustainable farming practices. The environmental implications of probiotics also merit attention. In aquaculture, the use of probiotics has been associated with a reduction in antibiotic residues and improved water quality, contributing to more sustainable farming practices [47]. Successful cases include the use of *Carnobacterium* and *Shewanella* probiotics in salmon to reduce *A. salmonicida* infections [48], and *Bacillus* or *Photobacterium* strains in marine fish to combat *Vibrio* spec [49]. For instance, supplementing *B. subtilis* in feed during vaccination led to up to 86% survival in European seabass challenged with *V. anguillarum*, versus much lower survival in fish that were only vaccinated without probiotics [50]. In such cases, probiotics enhanced mucosal immune responses, improving vaccine performance. One striking example of probiotic impact is seen when they are combined with vaccines, as probiotics can act synergistically with immunization to improve antigen uptake at gut-associated lymphoid tissue and boost specific antibody levels [51]. Delivery methods unique to aquaculture have also been explored, including bathing fish in probiotic-rich water or administering probiotics via live feed such as brine shrimp nauplii [44]. One striking example of probiotic impact is seen when they are combined with vaccines. A recent review highlighted that probiotics can act synergistically with fish vaccines: for instance, supplementing *B. subtilis* in feed during vaccination led to up to 86% survival in European seabass challenged with *V. anguillarum*, compared to much lower survival in fish that were only vaccinated without probiotics. Probiotics in that case enhanced mucosal immune responses, improving antigen uptake at gut-associated lymphoid tissue and boosting specific antibody levels, thereby significantly “improving vaccine performance”. Delivery methods unique to aquaculture have been explored too—including bathing fish in probiotic-rich water or even administering probiotics via live feed (e.g., brine shrimp nauplii carrying probiotics to larval fish). These methods can seed the fish’s microbiome early on, sometimes conferring disease resistance from fry stages [52]. In shrimp and other shellfish, probiotics are heavily used not only for the animal’s gut health but also for water quality management. Intensive shrimp farming often suffers from water quality deterioration and pathogen outbreaks (like *Vibrio harveyi* causing vibriosis or AHPND). Probiotics (commonly *Bacillus* spp., *Nitrobacter*, *Lactobacillus*, and *Photosynthetic* bacteria) are added to shrimp ponds to perform multiple roles. They “improve water microbial composition” and reduce ammonia and nitrite levels by promoting beneficial bacterial communities that degrade waste products [53,54,55]. Similarly, the inclusion of probiotics in livestock feed has been linked to decreased greenhouse gas emissions, particularly methane, as a result of altered rumen fermentation pathways [56]. Emerging research is also exploring the potential of probiotics to influence metabolic disorders in animals, including obesity and insulin resistance, through their impact on gut microbiota composition and energy regulation [46]. Also, Dandrieux (2016) [57] argues that “chronic enteropathy” is a more appropriate term than “inflammatory bowel disease” in dogs, as it encompasses a broader spectrum of conditions with variable responses to treatment—including dietary, antibiotic, and immunosuppressive therapies—distinct from human IBD in pathogenesis, classification, and clinical outcome [55]. Beyond scientific strategies such as microencapsulation and the use of prebiotics or synbiotics to enhance probiotic performance, successful application in practice also depends on regulatory requirements. Regulatory standards often require evidence of quality control: the strain must be deposited in a culture collection and designated with a unique identifier [56]. Codex Alimentarius guidelines on probiotics in food emphasize accurate labeling (the genus, species, and strain designation, along with viable count, should be on the label) and require that health claims be substantiated by scientific evidence. For animal probiotics, different jurisdictions have different requirements, but generally a demonstration of safety and some evidence of efficacy in target species is needed for approval. The European Union (EFSA) requires, for feed additives, a dossier including safety studies (e.g., showing no antibiotic resistance beyond intrinsic, no toxin genes) and at least three controlled efficacy studies in the target species demonstrating the claimed benefit [58]. Despite the promising benefits, challenges remain in their widespread adoption. These include variability in strain efficacy, lack of standardized dosing protocols, and gaps in understanding host-specific responses to different probiotics. Furthermore, regulatory frameworks governing probiotic use in animal feed vary widely, and approval often requires rigorous safety and efficacy data, which can be a barrier for commercialization. Economic constraints, particularly for small-scale producers, also hinder adoption if probiotic costs do not clearly justify the benefits.

## 2. Materials and Methods

This review synthesizes evidence from 197 peer-reviewed sources. A systematic search was conducted using PubMed, Scopus, Web of Science, and Google Scholar, with keywords such as ‘probiotics,’ animal gut microbiota, ‘livestock probiotics,’ and ‘companion animal health.’ Inclusion criteria required peer-reviewed articles focused on probiotic effects on animal health. Articles solely about human probiotics, were not excluded. The methodological quality of the included studies was assessed using established risk-of-bias tools, specifically RoB 2.0 for clinical trials and SYRCLE for animal studies, to ensure robust evaluation of the evidence. Boolean operators like AND and OR were applied to combine keywords effectively, ensuring the retrieval of relevant literature across diverse topics related to probiotics and animal health. Articles were included or excluded based on predefined criteria to maintain the quality and relevance of the review. Inclusion criteria required articles to be published in peer-reviewed journals, focused on the effects of probiotics on animal health, and written in English. Eligible articles included those discussing probiotic effects on gut health, immune modulation, disease resistance, or behavioral outcomes in livestock and companion animals. Studies published between 2000 and 2024 were prioritized to capture recent advancements. Exclusion criteria removed non-peer-reviewed materials, such as conference abstracts or editorials, as well as studies solely focused on probiotics for plants. Although the review primarily emphasizes livestock and companion animals, selected human studies were included when their findings were mechanistically relevant or extrapolable to veterinary medicine. Articles with unclear methodologies or insufficient results were also excluded. Data extraction focused on several key aspects. The most commonly studied probiotic strains in veterinary medicine extend beyond *L. acidophilus*, *B. longum*, and *S. boulardii*, and include species such as *L. reuteri*, *L. plantarum*, *E. faecium*, and *B. subtilis* in swine; *S. cerevisiae*, *B. licheniformis*, *P. freudenreichii*, and *L. casei* in ruminants; *L. johnsonii*, *L. salivarius*, *B. amyloliquefaciens*, and *E. faecium* in poultry; and *E. faecium*, *L. rhamnosus*, and *L. acidophilus* in dogs and cats. These strains were highlighted for their specific mechanisms of action and efficacy across different animal species. Target species examined in the reviewed studies included production animals such as swine, cattle, and poultry, as well as companion animals like dogs and cats. Key health outcomes were identified, including gut microbiota modulation, immune enhancement, pathogen exclusion, and improvements in stress-related behaviors. Many studies also explored the practical applications of probiotics, such as their use as antibiotic alternatives, growth promoters, and treatments for gastrointestinal disorders.

Each study was evaluated for transparency in methodology, sample size adequacy, accurate identification of probiotic strains, precise dosages, and statistical robustness. Randomized controlled trials (RCTs) were prioritized as they offered strong evidence of probiotic efficacy. The data collected from the reviewed studies were synthesized into thematic categories to identify trends and knowledge gaps. Probiotic mechanisms of action were a central theme, including their influence on gut microbiota composition, immune modulation, and pathogen inhibition. Livestock applications focused on probiotics as growth promoters, with significant evidence supporting their ability to improve feed conversion ratios and reduce pathogen loads. In companion animals, probiotics were shown to alleviate gastrointestinal issues, enhance immune responses, and reduce stress-related behaviors. Environmental benefits of probiotics were also noted, such as their potential to reduce methane emissions in ruminants and improve water quality in aquaculture systems. A meta-analysis was not conducted due to the heterogeneity observed in study designs, probiotic strains, dosages, and reported outcomes. This need has also been emphasized in the previous literature, where inconsistent methodologies were identified as major barriers to reproducibility and translation of probiotic research into veterinary and clinical practice [59,60]. In particular, a recent review by Marková et al. (2024) [61] exposed the misalignment between commonly targeted probiotic taxa and those microbial taxa actually correlating with improved performance in poultry, further underscoring methodological inconsistencies in the field [57].

### Methods for Figures

Figures were created using Bio Render (https://BioRender.com/55butv3, accessed date 15 June 2025).

## 3. Mechanisms of Action of Probiotics

Immunomodulatory effects are also strain-dependent; for instance, *L. reuteri* supplementation in pigs reduced pro-inflammatory cytokine expression and promoted regulatory T cell activity, highlighting probiotic influence on Th1/Th2/Th17 balance [62].

**Figure 2:**
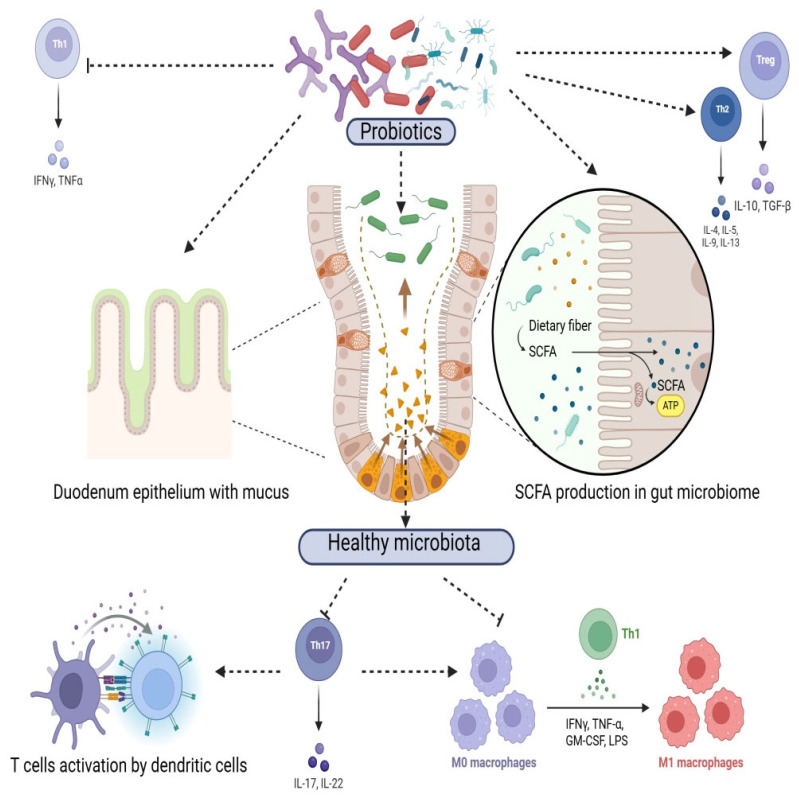
Immunity and Probiotics: The Gut Microbiota’s Function in Reducing Inflammation. Probiotics induce healthy gut microbiota. It can influence the immune response, as illustrated in this figure. Bacteria from the gut produce short-chain fatty acids (SCFAs), which have anti-inflammatory properties. This interaction reduces the pro-inflammatory activity of Th1 and Th17 cells while increasing regulatory T cells (Tregs) and anti-inflammatory cytokines (such as IL-10 and TGF-β). As a result, a balanced microbiome supports a robust immune system and helps avoid excessive inflammation.

Beyond gastrointestinal and immune-related effects, probiotics can also produce neuroactive compounds such as γ-aminobutyric acid (GABA), serotonin, and dopamine, which influence host physiology through the microbiota–gut–brain axis. This emerging field, termed microbial endocrinology, highlights that probiotics may act as delivery vehicles for neurochemicals, thereby linking gut microbiota composition to behavioral and neurophysiological outcomes in both livestock and companion animals [24,63]. Probiotics exert their beneficial effects on animal health through a range of complex mechanisms that target the gut microbiota, immune system, and host physiology. Reinforcement of epithelial barrier integrity is another important effect, as probiotics can up-regulate tight-junction proteins and reduce intestinal permeability, improving host resilience to infection [29,64]. One classical mechanism of action is competitive exclusion, where probiotics occupy ecological niches and compete for nutrients, thereby limiting pathogen colonization; in addition, several strains produce bacteriocins and organic acids that inhibit opportunistic microbes [65]. These mechanisms are highly strain- and species-specific, reflecting the diversity of probiotic organisms and their interactions with different host systems (Figure 2). Advances in encapsulation technologies have further enhanced the viability and efficacy of probiotics, ensuring their survival during processing, storage, and passage through the digestive tract [66]. Microencapsulation techniques, for instance, protect probiotics from harsh gastric conditions and improve their targeted delivery to the intestines [54]. These innovations have expanded the applicability of probiotics in both feed and pharmaceutical formulations [55]. Furthermore, the synergy between probiotics and prebiotics—collectively termed synbiotics—offers an additional layer of benefits by providing a substrate for beneficial bacteria, thereby enhancing their proliferation and activity within the gut. For example, combinations of *Lactobacillus* strains with dietary fibers have been shown to amplify both the microbial and host-derived benefits, such as increased short-chain fatty acid production and improved gut barrier function [67,68]. Furthermore, the synergy between probiotics and prebiotics—collectively termed synbiotics—offers additional benefits by providing a selective substrate for the probiotic organisms. For example, combinations of *Lactobacillus* strains with fermentable fibers have been shown to amplify beneficial outcomes, such as increased short-chain fatty acid production and improved gut barrier function [61,69]. Evidence from human studies also illustrates that probiotic efficacy depends on host-specific microbiome features; for instance, Maldonado-Gómez et al. (2016) [70] demonstrated that stable engraftment of *B. longum* AH1206 in the gut occurred only when compatible ecological niches were available in the host’s resident microbiota, highlighting the individualized nature of probiotic responses.

### 3.1. Gut Microbiota Modulation

One of the primary actions of probiotics is the modulation of the gut microbiota. Probiotic organisms compete with pathogenic bacteria for nutrients and adhesion sites on the intestinal epithelium, thereby reducing the colonization and proliferation of harmful microbes [4]. This process, known as competitive exclusion, has been demonstrated in livestock to reduce pathogens such as *Salmonella* and *Escherichia coli*. In swine, supplementation with *L. sobrius* and *E. faecium* has improved growth performance and stabilized fecal microbiota in weaned piglets [70]. In poultry, *B. subtilis* and *L. salivarius* strains have reduced necrotic enteritis and improved feed conversion efficiency [71]. In ruminants, *S. cerevisiae* enhances fiber digestibility, stabilizes rumen fermentation, and improves milk production efficiency, while *P. freudenreichii* contributes to rumen stability and nutrient utilization [72]. In companion animals, *E. faecium* and *L. acidophilus* have been shown to improve gastrointestinal health, reduce diarrhea episodes, and modulate fecal bacterial groups in dogs [73]. Additionally, probiotics enhance the abundance of beneficial bacterial taxa, including members of the genera *Lactobacillus* and *Bifidobacterium*, which play key roles in maintaining gut health [74]. Probiotics have also shown inhibitory effects against other harmful bacteria, including *Clostridium perfringens*, a major cause of necrotic enteritis in poultry and gastrointestinal disease in livestock. Additionally, probiotics enhance the abundance of beneficial bacterial taxa, including members of the genera *Lactobacillus* and *Bifidobacterium*, which play key roles in maintaining gut health [75].

Probiotics also influence microbial diversity and metabolic activity in the gut. For instance, certain strains stimulate the production of short-chain fatty acids (SCFAs) like acetate, propionate, and butyrate, which serve as energy sources for epithelial cells and contribute to maintaining intestinal integrity [76]. Increased SCFA production has been associated with improved gut barrier function and reduced inflammation in both livestock and companion animals [77].

Probiotic supplementation enhances the resilience of gut microbiota, allowing it to recover more effectively following disruptions such as antibiotic use, stress, or dietary changes. This resilience is pivotal in maintaining microbial homeostasis, which supports overall host health and productivity. Furthermore, probiotics influence inter-microbial communication via quorum sensing inhibition, which reduces pathogen virulence and biofilm formation, as demonstrated in various animal models [78]. Some strains also increase the production of bioactive metabolites, such as conjugated linoleic acids and antimicrobial peptides, which regulate inflammatory pathways and enhance nutrient uptake [79]. These multifaceted interactions underline the essential role of probiotics in fostering a stable and functionally diverse gut ecosystem, critical for optimal animal health and performance. Despite these benefits, probiotic responses are not universal. Outcomes may vary between individuals due to host genetics, diet composition, and baseline microbiota, and efficacy is often strain-specific [1,80].

### 3.2. Immune System Modulation

Another critical mechanism by which probiotics enhance health is through the modulation of the host immune system. Probiotics interact with gut-associated lymphoid tissue (GALT) to stimulate the production of immunoglobulins (e.g., IgA) and cytokines that regulate immune responses [81]. These interactions enhance mucosal immunity, providing a first line of defense against intestinal infections (Figure 2).

Probiotics can also modulate systemic immunity by balancing pro-inflammatory and anti-inflammatory pathways. For example, *L. reuteri* has been shown to reduce the levels of pro-inflammatory cytokines like tumor necrosis factor-alpha (TNF-α) while promoting the release of anti-inflammatory cytokines such as interleukin-10 (IL-10) [82]. This immunomodulatory effect is particularly beneficial in mitigating chronic inflammation and immune dysregulation in animals subjected to environmental or dietary stressors [83].

### 3.3. Pathogen Inhibition

Probiotic organisms produce antimicrobial compounds, including bacteriocins, hydrogen peroxide, and organic acids, which inhibit the growth of pathogenic bacteria [50]. These antimicrobial effects are strain-dependent and have been demonstrated in both in vitro and in vivo studies. For example, *Lactobacillus acidophilus* produces lactic acid, which lowers intestinal pH and creates an unfavorable environment for pathogens like *Clostridium perfringens* [24]. Many probiotic bacteria produce natural anti-microbials. Lactic acid bacteria (LAB) such as *Lactobacillus* and *Enterococcus* secrete organic acids (lactic, acetic acid) that lower gut pH, creating an unfavorable environment for pathogens (most pathogens grow poorly at low pH) [1]. LAB also produce bacteriocins, which are small peptide antibiotics capable of inhibiting or killing closely related bacterial species. For example, *L. salivarius* produces bacteriocins effective against *C. perfringens*, the causative agent of necrotic enteritis in poultry [84]. Similarly, *E. faecium* SF68, a probiotic used in pets, produces enterocins that inhibit *Salmonella* and *E. coli* in vitro [85]. These bacteriocins can reduce pathogen populations in the gut without the need for antibiotic intervention, thereby contributing to healthier microbial balance. These substances can reduce pathogen populations in the gut without the need for antibiotics. A practical illustration: a *Lactobacillus*-based probiotic culture given to chicks at 10^7 CFU per bird significantly reduced *Salmonella Enteriti* discounts in the ceca (as measured by culturing), indicating that the probiotic released inhibitory compounds or acids that curtailed Salmonella growth [85,86]. Some *Bacillus* probiotics used in livestock also secrete potent anti-microbials (e.g., subtilin, bacitracin-like lipopeptides) that suppress pathogens such as *Listeria* and *Staphylococcus*.

Additionally, probiotics can disrupt the quorum-sensing mechanisms of pathogenic bacteria, thereby inhibiting their ability to form biofilms and express virulence factors [86]. This property is particularly relevant in poultry farming, where biofilm-associated infections by *Salmonella* and *Campylobacter* pose significant challenges [87].

Probiotics influence the maturation and activity of innate immune cells, such as macrophages and neutrophils. They enhance macrophage phagocytosis, enabling more effective clearance of pathogens from the gut environment [88]. Certain probiotic strains, such as *L. rhamnosus*, are known to up-regulate the production of antimicrobial peptides, which act as a first line of defense against microbial invasion [83].

Additionally, probiotics interact with Toll-like receptors (TLRs), particularly TLR2 and TLR4, present on epithelial and immune cells. This interaction triggers intracellular signaling cascades, such as the NF-κB and MAPK pathways, leading to the secretion of cytokines that orchestrate immune responses [89]. This mechanism is particularly vital in modulating inflammation, as probiotics like *Bifidobacterium breve* balance pro-inflammatory and anti-inflammatory cytokines, helping to prevent overactivation of immune responses [90].

Probiotics further contribute to systemic immune modulation through the production of short-chain fatty acids (SCFAs), such as butyrate, which play a role in reducing inflammation and maintaining intestinal integrity. These SCFAs serve as energy sources for intestinal epithelial cells, promoting their proliferation and fortifying the mucosal barrier against pathogen translocation. Strains like *L. reuteri* are notable for stimulating regulatory T cells, which help maintain immune tolerance and reduce hypersensitivity to non-harmful antigens [91].

Finally, probiotics contribute to gut–brain axis communication, providing systemic benefits. By reducing stress-induced alterations in immune function, probiotics limit the impact of chronic stress on overall immunity [92]. These multifaceted mechanisms make probiotics indispensable tools in supporting balanced immune responses and improving resilience to both pathogenic challenges and environmental stressors (Figure 2).

### 3.4. Enhancement of Intestinal Barrier Function

Probiotics contribute to the maintenance of a healthy intestinal barrier by increasing the expression of tight junction proteins such as occludin and claudin [93]. These molecular pathways involve activation of protein kinase C (PKC), mitogen-activated protein kinase (MAPK), and modulation of toll-like receptor (TLR)-mediated signaling, which collectively stabilize tight junction complexes and limit paracellular permeability [94]. This barrier reinforcement prevents the translocation of pathogens and endotoxins into systemic circulation [95]. In young cattle, supplementation with *S. cerevisiae* has contributed to the prevention of neonatal diarrhea, partly through stabilization of the intestinal barrier and modulation of local immune responses [96]. This strengthens the epithelial barrier, preventing the translocation of pathogens and endotoxins into systemic circulation. Studies in swine have shown that probiotic supplementation reduces intestinal permeability and the associated risk of systemic infections during stressful periods like weaning [43].

In addition to their effects on tight junctions, probiotics also promote the secretion of mucus by goblet cells in the intestinal lining (Figure 2). This mucus layer acts as a physical barrier, trapping pathogens and facilitating their removal from the gut [31].

### 3.5. Mechanisms Linking Probiotics to Stress and Depression in Animals

Probiotics have emerged as a promising tool for modulating the gut–brain axis, which is a bidirectional communication network between the gastrointestinal tract and the central nervous system. This relationship highlights their potential to alleviate stress and depression through several mechanisms [32].

Firstly, probiotics such as *L. rhamnosus* can influence the production of neurotransmitters. For example, in a mouse model, *L. rhamnosus* was shown to alter gamma-aminobutyric acid (GABA) receptor expression in the brain, resulting in reduced anxiety- and depression-like behaviors. Evidence in other species suggests similar potential effects. In pigs, supplementation with *L. rhamnosus* and *B. longum* has been associated with reduced cortisol levels and calmer behavior under stress conditions [97,98]. In poultry, *B. subtilis* supplementation has been linked to lower stress indicators and improved welfare outcomes, including reduced feather-pecking [99,100]. In dogs, *E. faecium* has shown promise in supporting behavioral stability during stressful events, such as shelter housing and travel [74,101].

Secondly, chronic stress activates the hypothalamic–pituitary–adrenal (HPA) axis, resulting in elevated corticosterone levels that are associated with depressive behaviors. Probiotics can mitigate this response by reducing stress-induced increases in adrenocorticotrophic hormone and corticosterone, which alleviates anxiety and depression-like symptoms [38].

Thirdly, inflammation plays a significant role in the development of depression. Probiotics can reduce systemic inflammation by enhancing gut barrier function and modulating immune responses. Studies have shown that probiotic supplementation decreases levels of pro-inflammatory cytokines, which are often elevated in depressive states [82]. While much of the mechanistic detail comes from rodent models, these findings provide translational insight into potential applications for improving welfare in livestock and companion animals. More comparative studies are needed to confirm whether the same neuroendocrine and immunological pathways observed in mice are consistently modulated in veterinary species.

#### Probiotics and Cancer in Animals

Although direct research on the effects of probiotics on cancer-related depression in animals remains limited, their role in cancer progression and associated mechanisms provides insights into their potential benefits [102]. A robust immune system is vital for combating cancer, and probiotics enhance immune responses, potentially inhibiting tumor growth. Certain probiotic strains have been shown to increase the activity of natural killer cells and macrophages, which are critical components of the body’s defense mechanisms against cancer [103].

In addition to immune modulation, probiotics contribute to anticancer effects through several other mechanisms. They can induce tumor apoptosis and promote cell cycle arrest in malignant cells [104]. Probiotics also produce anticancer metabolites, including short-chain fatty acids (SCFAs) such as butyrate and propionate, which have been implicated in the inhibition of tumor cell proliferation and induction of apoptosis [105]. Conjugated linoleic acid (CLA), generated by certain *Lactobacillus* and *Propionibacterium* species, exhibits anticarcinogenic activity by modulating lipid metabolism and suppressing tumor growth [106]. Moreover, probiotics can beneficially modulate the intestinal microbiome, reducing procarcinogenic metabolites such as secondary bile acids and nitrosamines, thereby lowering cancer risk [107].

For instance, oral administration of *L. casei* variety *rhamnosus* (Lcr35) and a combination of *L. acidophilus* and *B. bifidum* (LaBi) in mice undergoing 5-FU chemotherapy resulted in decreased diarrhea severity, reduced pro-inflammatory cytokine levels, and improved intestinal histology [108]. By influencing the gut–brain axis, probiotics may indirectly impact cancer progression and associated mood disorders. Alterations in gut microbiota composition have been linked to changes in behavior and cognition in animals, suggesting a pathway through which probiotics could exert beneficial effects [109].

## 4. Applications of Probiotics in Animal Health

### 4.1. Livestock Animals

Probiotics have become a cornerstone of sustainable livestock farming, offering solutions for enhanced productivity, health, and environmental management. Applications of probiotics in animal health can be discussed across three categories: livestock animals (cattle, pigs, poultry, horses), companion animals (dogs, cats), and laboratory animals used as experimental models (rats)

#### 4.1.1. Cattle

In dairy cattle, probiotics can improve milk yield and composition. Inclusion of *S. cerevisiae* in feed stabilizes rumen pH and supports cellulolytic bacteria, which enhances fiber digestion and energy availability and in turn influences milk production and quality [110]. Probiotic use has also been associated with higher milk fat and protein, traits that are important for market value [111]. Probiotics help mitigate metabolic disorders such as ruminal acidosis that arise in high-concentrate diets. Lactate-producing bacteria such as *L. plantarum* and lactate-utilizing bacteria such as *P. freudenreichii* can promote conversion of lactate to propionate and thereby stabilize rumen pH while improving energy metabolism. Environmental benefits are increasingly reported [112]. Certain probiotics, including *B. subtilis*, are associated with lower enteric methane output by redirecting hydrogen away from methanogenesis. This reduces energy loss as methane and can modestly improve feed efficiency [113]. Responses differ across production systems and physiological stages. In dairy herds, probiotics may enhance feed utilization and milk production, although results vary by strain, dose, and diet. For example, selected lactate-producing bacteria have improved milk yield and feed efficiency in some trials, likely through pH stabilization and improved fiber digestion [114]. A meta-analysis in dairy calves reported increases in pre-weaning average daily gain of approximately 40 to 80 g per day and better feed conversion ratio, while also noting inconsistent or contradictory findings in some studies [115]. In beef feedlot cattle, direct-fed microbials (DFM) are used primarily to support growth and carcass safety. Reviews report modest improvements in average daily gain (about 0.08 kg) and feed conversion ratio (about 0.13) under intensive conditions [116]. Beyond performance, probiotics can reduce pathogen shedding. For example, feeding specific *Lactobacillus* strains to feedlot cattle before slaughter reduced fecal shedding of *E. coli* O157:H7 and shortened the duration of carriage; by the end of one trial, none of the probiotic-treated steers were shedding O157 in the rumen compared with high prevalence in controls [117]. These effects have downstream implications for food safety. Production context shapes cost-effectiveness. Under extensive grazing, a diverse rumen microbiota may limit marginal gains, whereas in high-grain feedlots with greater risk of subacute ruminal acidosis, probiotics tend to be more beneficial. Economic analyses consider improvements in feed efficiency and health outcomes against product cost, with positive returns contingent on strain efficacy and pricing [55].

#### 4.1.2. Poultry

The poultry industry faces persistent challenges related to gut health, pathogen control, and feed efficiency. In ovo administration of *B. bifidum* and *B. longum* on day 17 of incubation improved broiler growth and ileal development without adverse effects on serum biochemistry or hepatic and renal indicators [118]. Probiotics address production constraints through complementary mechanisms that include modulation of the gut microbiota, enhancement of nutrient absorption, reinforcement of barrier function, and support of mucosal and systemic immunity. Intestinal morphology and barrier integrity are central to performance outcomes. In broilers, *L. reuteri* and *B. bifidum* increase villus height and the villus-to-crypt depth ratio, which improves nutrient absorption and feed conversion ratio (FCR), yielding higher body weight gains and lower feed costs [84]. Pathogen control remains a priority for both animal health and food safety. Probiotics limit colonization by *Salmonella* spp. and *Campylobacter* spp. through competitive exclusion and through production of bacteriocins, organic acids, and hydrogen peroxide that create inhibitory conditions for enteric pathogens [119]. These reductions are relevant to lowering slaughter-phase prevalence. Probiotics offer practical alternatives to antimicrobial growth promoters (AGPs) in both meat and egg production. In commercial broilers, *B. subtilis* has improved growth performance and reduced mortality [100]. In laying hens, probiotic use has been associated with lower circulating pro-inflammatory cytokines (IL-1, IL-6, TNF-α) and higher serum antioxidant enzyme activity, suggesting mitigation of systemic inflammation and oxidative stress in aging flocks [120]. In challenge models, a multi-species probiotic containing *Lactobacillus* and *Bacillus* hastened clearance of *S. Enteritidis*, with a markedly higher proportion of cleared birds by day 28 compared with untreated controls, and with performance similar to an oxytetracycline reference group [119]. Supplementation with *L. rhamnosus* has also reduced cecal *Salmonella* by approximately 2 log CFU in treated broilers [119]. *B. subtilis* strengthens the intestinal barrier and immunity by up-regulating tight-junction proteins, cytokines, and immunoglobulins, with dual-strain formulations showing consistent effects [120]. Under heat stress, combinations of *B. subtilis* with *P. farinosa* or *Lactobacillus* spp. improve feed efficiency, lower pathogenic *E. coli*, and favor beneficial taxa [121]. A recent broiler trial also reported that dietary *B. xiamenensis* increased final body weight, improved villus morphology, and reduced cecal *E. coli* and *Salmonella* counts relative to controls [122]. Drinking-water supplementation with *B. subtilis* and *B. pumilus* mitigates heat-stress damage by partially restoring jejunal and ileal villus height and improving thermoregulation indices [123,124].

#### 4.1.3. Swine

Probiotics play a pivotal role in swine production, particularly during the critical post-weaning period. Weaning is associated with significant stress, gut microbiota disruption, and increased susceptibility to diarrhea, all of which adversely affect growth performance. *L. rhamnosus* and *B. lactis* have demonstrated efficacy in restoring microbial balance during the post-weaning period, reducing the prevalence of *Escherichia coli*-associated diarrhea [125]. These probiotics enhance intestinal barrier integrity by increasing tight junction protein expression and preventing the translocation of pathogens and toxins [126]. Probiotic supplementation also improves nutrient digestibility and growth performance in pigs. By increasing the production of short-chain fatty acids (SCFAs), probiotics stimulate epithelial cell proliferation and energy utilization in the gut [89]. Enhanced digestion and absorption of nutrients translate to higher daily weight gains and better feed efficiency. In addition to gastrointestinal benefits, probiotics modulate systemic immunity in swine. Probiotic-fed pigs exhibit increased serum levels of immunoglobulins (IgA and IgG) and reduced pro-inflammatory cytokines, indicating enhanced immune resilience [127]. These effects are particularly valuable in intensive farming systems, where disease outbreaks pose significant economic risks. In swine, probiotics are actively studied as alternatives to AGPs, with applications from sows through nursery and finishing pigs [13,128]. With many countries phasing out in-feed antibiotics, interest in probiotics for herd health and growth has surged. In gestating and lactating sows, probiotics have shown benefits for both sow well-being and offspring performance. A recent controlled study in late-gestation sows found that a *Bacillus*-based probiotic significantly relieved constipation and systemic inflammation in sows, and improved piglet growth. Treated sows had higher anti-inflammatory cytokines (IL-4, IL-10) and lower pro-inflammatory markers (IL-1β, TNF-α), indicating reduced inflammation. Notably, their piglets had higher daily weight gain and weaning weights compared to controls [13,127]. Other trials report that feeding sows probiotics (e.g., *C. butyricum* or multi-strain blends) during late gestation/lactation can reduce neonatal diarrhea in piglets by improving colostrum quality and seeding a healthier microbiome in the neonate. In a comprehensive review, Su et al. (2022) [13] highlight that post-weaning piglets suffer from disrupted microbial homeostasis and compromised intestinal barrier function, features often addressed by antibiotics, and that probiotics can restore microbial balance and reinforce intestinal mucosal and immunological barriers to help prevent post-weaning diarrhea (PWD). Trials have shown that piglets given probiotic supplements experience lower incidence of PWD and improved weight gains compared to unsupplemented controls. Sows on probiotics often show improved feed intake, fewer peripartum digestive upsets, and possibly improved mood/behavior—one study observed probiotic-supplemented sows were calmer and their piglets less aggressive, hinting at gut–microbiota effects on behavior [13,129]. For piglets, probiotics are chiefly used to prevent post-weaning diarrhea (PWD) and improve growth in the nursery phase. Early weaned piglets undergo stress and gut microbiota disruption, traditionally managed with antibiotics [130]. Probiotics offer a promising alternative. Multiple studies document that certain probiotic strains can avert and treat PWD by reinforcing the intestinal barrier and immune defenses. For example, *Lactobacillus* and *Enterococcus* strains have been shown to reduce the incidence and severity of PWD. In one trial, piglets given a mix of *L. acidophilus*, *L. casei*, *B. thermophilum*, and *E. faecium* had a >50% reduction in diarrhea incidence post-weaning [131].

#### 4.1.4. Probiotics in Horses

Horses have a specialized hindgut microbiota that is essential for fermenting fiber and maintaining overall health. Disruptions to this microbial ecosystem, often caused by dietary changes or stress, can lead to conditions such as colic, laminitis, and diarrhea [132] (Table 1). Probiotics provide a natural solution to stabilize the equine gut and prevent these serious health issues [133]. Colic is among the most common and potentially life-threatening conditions in horses. Probiotic strains such as *Saccharomyces boulardii* have been shown to stabilize the hindgut microbiota, limiting the proliferation of gas-forming bacteria and thereby reducing gastrointestinal disturbances. This protective effect is particularly valuable during dietary transitions, which are recognized risk factors for digestive upset and colic [134]. Probiotics also play a role in preventing laminitis, a painful and often debilitating condition associated with hindgut acidosis. By improving the fermentation of dietary fibers and reducing the accumulation of lactic acid, probiotics help maintain a stable hindgut environment. Strains such as *L. plantarum* are effective in mitigating the cascade of events that lead to laminitis, particularly during sudden dietary changes, when horses consume diets high in starch or fructans, such as excess grain or lush spring pastures, undigested carbohydrates reach the hindgut, where amylolytic bacteria, such as streptococci, rapidly ferment them, producing excess lactic acid. This causes a sharp drop in hindgut pH, leading to acidosis that disrupts the microbial balance by killing off beneficial fibrolytic bacteria. *Lactobacillus plantarum* intervenes by competing with these amylolytic bacteria for carbohydrate substrates, slowing their fermentation and reducing the accumulation of lactic acid. In ex vivo studies, *L. plantarum* has been shown to limit pH drops by up to 1 unit during starch overload, helping to maintain a more stable environment in the hindgut. This action attenuates the initial dysbiosis that sets the stage for laminitis, making *L. plantarum* a key ally in the prevention of diet-induced laminitis [135]. Horses often experience stress during transport or competition, which can lead to gut dysbiosis and associated health issues. Probiotics enhance gut barrier integrity and reduce inflammatory cytokines, mitigating the effects of stress on the gut. In equine trials, supplementation has been associated with stabilized fecal pH, reduced lactic acid accumulation, and improved volatile fatty acid profiles following stressors such as transport, supporting a protective role against dysbiosis [74]. For adult horses, probiotics have been considered for various purposes: maintaining gut health in performance horses, preventing colic and laminitis, and aiding recovery from colitis [136]. Horses are hindgut fermenters highly susceptible to dysbiosis when diet or routine changes (e.g., high starch intake, sudden feed change). Probiotics, especially yeast cultures and lactic acid bacteria, have been given to stabilize the hindgut pH and microbial balance [137]. Some studies with performance horses (e.g., racehorses or events under intensive training) indicate probiotics may reduce stress-related gut disturbances. One study found that horses undergoing transport-like stress had more stable fecal microbiota when supplemented with a yeast-derived post-biotic (SCFP), compared to controls, though effects on stool consistency were not reported [138]. Additionally, an intriguing line of research is linking the gut microbiome with horse behavior and stress. As with other species, identifying the right strains for the equine gut and ensuring they reach the hindgut alive (many bacteria may be digested before reaching the cecum) are ongoing challenges. In terms of laminitis, which is often precipitated by hindgut dysbiosis (excess starch in the hindgut causing a bloom of lactic-acid bacteria and toxin release), there is theoretical rationale to use probiotics to prevent those damaging imbalances. Researchers have identified certain fiber-digesting bacteria that decline during laminitis episodes and hypothesize that replenishing them could avert the cascade leading to laminar inflammation [62]. Although disturbances in fiber-digesting bacteria have been implicated in laminitis pathophysiology, there is currently no clinical trial evidence that probiotic administration prevents this condition. At best, some probiotics might reduce the risk by maintaining a healthier hindgut environment when horses consume high carbohydrate diets—this is an active area of investigation [139]. Until such data is available, the use of probiotics in horses will likely remain cautious and adjunctive—an optional tool in the toolbox rather than a mainstay therapy.

### 4.2. Laboratory Animals

Experimental studies involving rats provide crucial insights into the mechanisms and potential benefits of probiotics. Rats are widely used in research due to their controlled environments and genetic homogeneity, which enable detailed investigations into the physiological and biochemical effects of probiotics. These studies have advanced our understanding of probiotics’ roles in gastrointestinal health, immune modulation, and systemic well-being [140]. Probiotics in rats primarily act by modulating gut microbiota composition and function. They enhance the production of short-chain fatty acids (SCFAs) such as butyrate, which strengthens the intestinal barrier and supports epithelial cell health [141]. In a murine model, the administration of a probiotic mixture containing *L. acidophilus* and *B. longum* significantly enhanced mucosal immunity by increasing the number of IgA-producing cells in Peyer’s patches and the lamina propria. Furthermore, the same study demonstrated a marked reduction in pro-inflammatory cytokines, including IL-6, IL-12, and TNF-α, highlighting the immunomodulatory potential of these probiotics [142]. These effects collectively contribute to improved gut integrity and reduced inflammation. Rats have been instrumental in demonstrating the diverse health benefits of probiotics. For gastrointestinal health, probiotics mitigate the severity of experimental colitis, enhance gut barrier function, and alleviate symptoms of irritable bowel syndrome (IBS) [143]. Systemically, probiotics influence metabolic conditions by improving insulin sensitivity and reducing markers of inflammation, suggesting their role in managing obesity and related disorders [144]. Additionally, probiotics have shown promising effects on the gut–brain axis in rat models, reducing anxiety-like behaviors and other stress-induced conditions through microbiota-mediated mechanisms [145]. The findings from studies in rats have significant implications for both human and animal health. These models highlight the therapeutic potential of probiotics in treating chronic gastrointestinal disorders, enhancing systemic immunity, and even addressing neurobehavioral conditions. As research advances, precision probiotics tailored to target specific pathways and conditions may emerge, providing enhanced efficacy and specificity [146]. Experimental studies involving rats provide crucial insights into the mechanisms and potential benefits of probiotics. Rats are widely used in research due to their physiological similarities to other mammals and controlled experimental tractability.

### 4.3. Companion Animals

Probiotics have become increasingly popular in the care of companion animals, including dogs, and cats. These animals frequently encounter health challenges such as gastrointestinal disturbances, immune system dysfunctions, and stress-induced conditions, all of which probiotics can help address. By targeting the gut microbiota, probiotics contribute to enhanced health and well-being in these species.

#### 4.3.1. Dogs

Dogs are particularly susceptible to gastrointestinal disorders, including acute diarrhea, chronic enteropathies, and inflammatory bowel disease (IBD). Probiotic supplementation has demonstrated significant benefits in managing these conditions by restoring microbial balance and improving gut barrier integrity [147]. For example, strains such as *L. acidophilus* and *E. faecium* have been shown to reduce the duration and severity of diarrhea in dogs by suppressing pathogenic bacteria like *C. difficile*. These probiotics also improve stool quality, providing relief for dogs with acute or chronic gastrointestinal distress [148]. In addition to gut health, probiotics play a vital role in modulating the canine immune system. By enhancing the production of secretory immunoglobulin A (IgA) in the gut, probiotics strengthen mucosal immunity, making dogs more resilient to infections and illnesses. This effect is particularly valuable for dogs recovering from illness or undergoing antibiotic therapy, where gut microbiota can be compromised [149]. Probiotics also impact canine behavior through the gut–brain axis. Research has shown that strains like *L. rhamnosus* produce neuroactive compounds that interact with the vagus nerve, influencing stress and anxiety levels [150]. A large double-blind, randomized, placebo-controlled trial in shelters showed that dogs receiving a synbiotic containing *E. faecium* NCIMB 10415 experienced significantly fewer days with diarrhea compared to placebo-treated dogs, demonstrating the probiotic’s efficacy in managing acute gastrointestinal disturbances [151]. Dogs supplemented with these probiotics exhibit reduced stress-induced behaviors, particularly in situations involving environmental changes or separation anxiety. This emerging area highlights the potential of probiotics as a natural tool for managing behavioral issues in dogs [14]. Controlled clinical trials in dogs with acute diarrhea have yielded promising results. One randomized, placebo-controlled trial showed that dogs with acute diarrhea receiving a multi-strain probiotic had a faster return to normal stool (3.5 days on average) compared to 4.6 days in dogs treated with the antibiotic metronidazole [152]. In fact, the probiotic was statistically as effective as the antibiotic in resolving diarrhea, highlighting its potential as a first-line therapy for uncomplicated diarrhea. Another placebo-controlled study in shelter dogs found that a synbiotic (probiotic + prebiotic) significantly decreased the incidence of stress-related diarrhea—only 7.7% of probiotic-treated dogs had ≥2 days of diarrhea, versus 20.7% in the placebo group [153]. These findings suggest probiotics can help prevent diarrhea in high-stress environments (kennels, shelters) by mitigating dysbiosis. Probiotics have also been evaluated in dogs with chronic gastrointestinal diseases like Inflammatory Bowel Disease (IBD). A small study using the probiotic mixture VSL#3 in canine IBD showed reduced clinical severity and improved histologic scores, alongside increased expression of tight junction proteins (indicating improved gut barrier). While sample sizes are limited, such trials demonstrate tangible benefits of probiotics in restoring gut health [154]. Notably, in canine atopic dermatitis, two months of *L. sakei* Probio-65 administration significantly reduced disease severity as measured by the Pruritus Visual Analog Scale and the Canine Atopic Dermatitis Extent and Severity Index [155].

#### 4.3.2. Cats

In cats, gastrointestinal health is often disrupted by dietary sensitivities, stress, or medical treatments such as antibiotics [154]. Probiotics offer a natural means of improving gut health and overall well-being in felines. *B. animalis* may facilitate peptide assimilation via intracellular peptidases (e.g., PepO) and by reshaping colonic fermentation toward SCFA production; in cats, these shifts have been associated with improved barrier function and lower inflammatory tone [21]. *Lactiplantibacillus plantarum* increased serum IgA and IL-4, reduced TNF-α, and lowered circulating D-lactate and diamine oxidase (barrier injury markers), consistent with improved gut barrier integrity and an anti-inflammatory shift [156]. Probiotic supplementation has also been associated with improved stool consistency in cats with sensitive stomachs [157]. Probiotics have shown promise in managing conditions beyond the gastrointestinal tract, such as food allergies and skin disorders. Cats with allergic dermatitis often experience systemic inflammation, which can be alleviated through probiotic supplementation. In feline allergic dermatitis (FASS), cutaneous OSMR-β (part of the IL-31 receptor complex) is significantly up-regulated in lesional skin, whereas classical Th2 cytokines are low/variable—underscoring that IL-4/IL-13-centric paradigms from dogs/humans do not transfer wholesale to cats [40,158]. By modulating the immune response, probiotics reduce inflammation, improving skin health and coat quality. This dual benefit of improved gut and skin health underscores the systemic effects of probiotics in felines [15]. Behavioral benefits of probiotics are also being explored in cats. Research suggests that probiotics such as *L. reuteri* can reduce signs of stress during transport or veterinary visits. This calming effect is thought to be mediated by the gut–brain axis, similar to findings in dogs. Probiotics could therefore play a role in reducing stress-related health issues in cats [74,147]. Mechanistically, *B. animalis* AHC7 attenuates NF-κB activation in vivo, decreases TNF-α/IFN-γ from Peyer’s patch lymphocytes, and, via dendritic-cell conditioning, expands CD25^+^Foxp3^+^ regulatory T cells, offering a plausible Treg-mediated route by which bifidobacteria dampen mucosal inflammation (cross-species data supporting a conserved pathway) [159]. Nonetheless, a few controlled trials in cats have been conducted, mostly for diarrhea management. In one study, older shelter cats were given *E. faecium* SF68 (a common pet probiotic) to see if it would prevent stress diarrhea; it did not significantly prevent diarrhea occurrence, but it did decrease the number of cats with prolonged diarrhea (≥2 days). In shelter cats, *E. faecium* SF68 reduced prolonged diarrhea episodes, with a lower proportion experiencing ≥ 2 days of diarrhea compared to controls [160,161].

**Table 1:** Probiotics employed in different animal species and their reported effects.

Animal Species	Probiotic Strains/Formulations	Reported Effects	References
Cattle (Dairy and Beef)	*Saccharomyces cerevisiae*	Stabilizes rumen pH, supports cellulolytic bacteria, improves fiber digestion, enhances milk yield and composition	[109,110]
	*Lactobacillus plantarum*, *Propionibacterium freudenreichii*	Converts lactate to propionate, stabilizes rumen pH, improves energy metabolism	[111,112]
	*Bacillus subtilis*	Reduces enteric methane emissions, improves feed efficiency	[113]
	Lactate-producing bacteria (various strains)	Improve milk yield and feed efficiency by stabilizing rumen pH	[114]
	*Lactobacillus* spp. (specific strains)	Reduces fecal shedding of *E. coli* O157:H7 in feedlot cattle, improving food safety	[116]
Poultry (Broilers and Layers)	*Bifidobacterium bifidum*, *B. longum* (in ovo)	Improves broiler growth and ileal development	[118]
	*Lactobacillus reuteri*, *B. bifidum*	Increase villus height and villus/crypt ratio, improve nutrient absorption and feed conversion	[162]
	*Bacillus subtilis*	Improves growth performance, reduces mortality, strengthens intestinal barrier	[119,120]
	Multi-species mix (*Lactobacillus*, *Bacillus*)	Hastens clearance of *Salmonella Enteritidis*	[118]
	*Lactobacillus rhamnosus*	Reduces cecal *Salmonella* (~2 log CFU reduction)	[118]
	*Bacillus xiamenensis*	Increases body weight, improves villus morphology, reduces *E. coli* and *Salmonella*	[122]
	*B. subtilis*, *B. pumilus* (water)	Mitigates heat stress, restores villus height	[122]
Swine (Sows & Piglets)	*Lactobacillus rhamnosus*, *Bifidobacterium lactis*	Restores microbial balance post-weaning, reduces *E. coli* diarrhea	[127]
	*Clostridium butyricum* (multi-strain blends)	Improves colostrum quality, reduces neonatal diarrhea	[127]
	*Bacillus*-based probiotics	Relieve constipation and systemic inflammation in sows, improve piglet growth	[127]
	Multi-strain mix (*L. acidophilus*, *L. casei*, *B. thermophilum*, *E. faecium*)	Reduces post-weaning diarrhea incidence by >50%	[131]
Horses	*Saccharomyces boulardii*	Stabilizes hindgut microbiota, reduces gas-forming bacteria, prevents colic	[133]
	*Lactobacillus plantarum*	Reduces hindgut lactic acid accumulation, stabilizes pH, mitigates laminitis risk	[135]
	Yeast cultures (e.g., *S. cerevisiae* post-biotics)	Stabilize fecal microbiota during stress/transport	[138]
Laboratory Animals (Rats)	*Lactobacillus acidophilus*, *B. longum*	Enhance mucosal immunity (↑IgA), reduce pro-inflammatory cytokines, improve gut integrity	[143,145]
Dogs	*Lactobacillus acidophilus*, *Enterococcus faecium*	Reduce diarrhea severity/duration, improve stool quality, strengthen mucosal immunity	[146,148,149]
	*Lactobacillus rhamnosus*	Produces neuroactive compounds influencing stress/anxiety	[101,151]
	Multi-strain probiotic	Faster resolution of acute diarrhea, comparable to metronidazole	[151,156]
	Synbiotic (*E. faecium* NCIMB 10415 prebiotic)	Reduces stress-related diarrhea in shelters	[146,148]
	VSL#3 (multi-strain)	Reduces clinical severity of IBD, improves gut barrier	[151]
	*Lactobacillus sakei* Probio-65	Reduces severity of atopic dermatitis	[14]
Cats	*Bifidobacterium animalis*	Improves SCFA production, enhances barrier function, lowers inflammation	[150,154]
	*Lactiplantibacillus plantarum*	Increases IgA/IL-4, reduces TNF-α, improves gut barrier	[154]
	*Lactobacillus reuteri*	Reduces stress during transport or vet visits	[158]
	*Enterococcus faecium* SF68	Decreases prolonged diarrhea episodes in shelters	[21,155]

Legend: ↑—enhance immunity by increasing IgA.

## 5. Environmental and Sustainability Aspects of Probiotics

Probiotics have transformative potential beyond improving animal health. Their applications align closely with goals for environmental sustainability, offering practical solutions to reduce pollution, mitigate greenhouse gas emissions, enhance resource efficiency, and combat the global issue of antimicrobial resistance. These benefits position probiotics as a cornerstone of sustainable animal agriculture. Recent studies also emphasize that probiotic interventions can lower methane emissions in ruminants by shifting fermentation toward propionate production, thereby reducing the carbon footprint of livestock production [163]. Meta-analysis of probiotic interventions shows that feeding direct-fed microbials to cattle can reduce ruminal methane emissions by approximately 5–15%, depending on diet and strain selection. Meta-analyses indicate that, overall, probiotic (direct-fed microbial) supplementation does not reliably lower enteric methane in cattle; any reductions appear strain-, diet- and duration-dependent and are generally small at the aggregate level [164]. Recent reviews highlight that probiotics may serve as viable alternatives to antibiotics in livestock production, with some evidence suggesting they can reduce antibiotic usage and influence the resistome, thereby helping to limit environmental dissemination of resistance genes [165].

### 5.1. Reducing Methane Emissions in Livestock

Ruminants like cattle and sheep produce significant amounts of methane, a potent greenhouse gas, during rumen fermentation. Methane accounts for up to 10–12% of the gross energy loss in ruminants, making its mitigation both an environmental and an economic priority [62] (Table 2). Probiotics such as *S. cerevisiae* and *B. subtilis* reduce methane emissions by altering microbial populations in the rumen [166]. These probiotics reduce rumen protozoa and the protozoa-associated methanogenic archaea (PAM), thereby contributing to lower methane production [76]. VFAs serve as an energy source for the animal, increasing feed efficiency while reducing methane output [158]. For example, studies have shown that dairy cows supplemented with *S. cerevisiae* emit 15–20% less methane while showing a 5% improvement in milk production [167]. Additionally, probiotics (e.g., *Saccharomyces cerevisiae* CNCM I-1077 and selected lactic acid bacteria) stabilize rumen pH—raising mean pH (6.53 vs. 6.32), increasing minimum pH (5.97 vs. 5.69), and reducing time spent below the SARA threshold (pH < 5.6)—and enhance the establishment of cellulolytic bacteria; these shifts increase fermentation efficiency and are associated with lower methane formation (e.g., 18–30.6% lower CH_4_ in vitro with *Lactobacillus plantarum* supernatant; >40–50% lower CH_4_ in vitro with methanotroph-based DFMs) [168]. This dual benefit underscores the potential of probiotics to make livestock farming more sustainable.

### 5.2. Improving Water Quality in Aquaculture

Aquaculture systems often suffer from water pollution caused by uneaten feed, animal waste, and the proliferation of pathogenic bacteria. These factors can lead to poor water quality, disease outbreaks, and environmental degradation in surrounding ecosystems [169]. Probiotics offer a natural solution to these challenges by improving both water quality and animal health [44]. Probiotic strains such as *B. subtilis* and *L. plantarum* are used in shrimp and fish farming to suppress harmful bacteria like *Vibrio* spp. Consistent with controlled trials synthesized in a recent meta-analysis, the overall pooled effect of probiotics on enteric methane was not significant; however, multi-strain bacterial probiotics reduced methane emissions (SMD = −0.36; 95% CI −0.62 to −0.11; *p* = 0.005), and consortia favoring reductive acetogenesis/propionate production showed a larger reduction (SMD = −0.71; 95% CI −1.04 to −0.36; *p* = 0.001); longer supplementation durations further strengthened these reductions [170]. In addition to disease control, probiotics contribute to better nutrient cycling. For instance, *B. subtilis* enhances the breakdown of organic matter, reducing ammonia and nitrate concentrations in water. For example, B. subtilis DM115 achieved 98.45% NH_3_–N removal within 24 h under optimized conditions; in field ponds, a Bacillus-based water probiotic (incl. B. subtilis) lowered unionized NH_3_ from 0.050 → 0.006 mg L^−1^ (T2) and 0.030 → 0.005 mg L^−1^ (T3) over 8 weeks—77–81% lower than the post-treatment control (0.026 mg L^−1^); differences were significant (ANOVA, different-letter superscripts; *p* ≤ 0.05). Additionally, an immobilized B. subtilis strain (sp. N4) optimized for aquaculture showed ~99% nitrite removal and was applied to keep ammonia/nitrite/nitrate low in intensive systems [171,172]. Lower ammonia levels improve water quality and create a healthier environment for aquatic animals [173]. Field studies have demonstrated that shrimp farms using probiotics experience a 20% reduction in mortality rates and a 30% improvement in growth rates compared to conventional systems [113]. Furthermore, these systems require fewer chemical treatments, reducing environmental risks associated with antibiotic and pesticide residues.

### 5.3. Reducing Antibiotic Usage

Antibiotics have long been used in animal agriculture for growth promotion and disease prevention. Globally, bacterial antimicrobial resistance (AMR) was associated with 4.95 million deaths (95% UI 3.62–6.57) in 2019, including 1.27 million deaths directly attributable to resistance. Economically, unchecked AMR is projected to reduce annual global GDP by 1.1–3.8% by 2050, with output losses reaching US$3.4 trillion per year by 2030, ~US$6.1 trillion by 2050, and pushing >28 million additional people into extreme poverty by 2050; healthcare expenditures in the high-AMR case reach ~US$1.2 trillion in 2050. In food-animal production, antibiotic use remains substantial—~99,502 tons in 2020, projected to 107,472 tons by 2030 (+8.0%), consistent with earlier estimates of a ~67% increase from 2010 to 2030 [174,175]. However, their overuse has led to the global crisis of antimicrobial resistance (AMR), which poses significant risks to human and animal health. Probiotics offer a viable alternative to antibiotics, particularly in intensive farming systems where disease pressures are high [176]. Probiotics enhance gut health by promoting a stable and diverse microbial community that naturally suppresses pathogens. Mechanisms such as competitive exclusion, production of bacteriocins, and immune modulation reduce the need for antibiotics to prevent or treat infections [132]. For example, in poultry farming, probiotics have been shown to prevent necrotic enteritis, a common bacterial disease caused by *C. perfringens*, with efficacy comparable to antibiotic treatments [177]. Beyond disease prevention, probiotics improve growth performance by enhancing nutrient absorption and FCR. In swine, studies have demonstrated that probiotic supplementation reduces the incidence of post-weaning diarrhea by 30% while improving daily weight gain by 10% [178]. These results highlight probiotics’ potential to replace antibiotics as growth promoters without compromising productivity. Reducing antibiotic use also mitigates the spread of antibiotic-resistant genes in agricultural systems. This aligns with global initiatives like the World Health Organization’s (WHO) Global Action Plan on antimicrobial resistance (AMR), which advocates for sustainable alternatives to antibiotics in animal farming [179].

### 5.4. Sustainable Farming Practices

Probiotics contribute to sustainable farming by improving resource efficiency, reducing waste, and enhancing overall productivity. One of the most significant benefits of probiotics is their ability to optimize nutrient utilization, leading to lower feed requirements per unit of production. For example, poultry-fed diets supplemented with probiotics show a 10% improvement in FCR, reducing the need for grain and other feed inputs [180]. Probiotics also reduce environmental pollution by decreasing nutrient excretion in manure. Nitrogen and phosphorus runoff from livestock operations are major contributors to water pollution and eutrophication. Probiotic supplementation reduces the excretion of these nutrients by improving their absorption in the gut. Studies in swine have shown that probiotic-fed pigs excrete 20% less nitrogen and phosphorus compared to non-supplemented controls [181]. In organic farming systems, probiotics align with principles of reduced chemical inputs and natural disease management. These systems often incorporate probiotics as part of integrated pest and disease control strategies. Additionally, the use of probiotic-treated manure as an organic fertilizer enhances soil health, contributing to circular farming systems where waste products are repurposed as valuable resources. Quantitatively, *Bacillus clausii* administration for one month in humans modulated 423 mucosal genes (158 up-regulated; 265 down-regulated) in duodenal biopsies. In human PBMC-derived dendritic cells, *Lactobacillus rhamnosus* Lcr35 produced dose-dependent transcriptional shifts: at MOI 0.01, 58 genes were up-regulated and 138 down-regulated, whereas at MOI 10, 823 genes were up-regulated and 859 down-regulated (≥3-fold change). qRT-PCR confirmed marked increases at MOI 10 in key inflammatory transcripts—CCL20 (~100×), IL1B (~300×), IL12B (~400×), and TNF-α (~200×) [182]. Probiotics also play a role in enhancing animal welfare, which is increasingly prioritized in sustainable farming practices. By reducing the prevalence of disease and improving overall health, probiotics contribute to better living conditions for animals, aligning with consumer demands for ethically produced animal products.

### 5.5. Molecular Mechanisms of Probiotic Action

Multiple mechanisms underlie these gene-level effects. Some probiotic cell components (like peptidoglycans, teichoic acids, or DNA rich in CpG motifs) act as ligands for host receptors (TLRs, NOD-like receptors), thereby activating signaling pathways (MAPK, NF-κB, etc.) in immune cells. The outcome can be cell-type specific: for example, probiotics interacting with dendritic cells can cause up-regulation of IL-10 andIL-12 genes, skewing T cell responses toward regulatory or Th1 phenotypes depending on context [183]. In epithelial cells, probiotics have been noted to activate pathways that strengthen cell junctions one strain of *Lactobacillus* was shown to increase phosphorylation of occludin and ZO-1 (tight junction proteins), thereby reinforcing the gut barrier at the protein level. Another molecular action is the secretion of metabolites by probiotics that then act as signaling molecules. Short-chain fatty acids (SCFAs) like butyrate, produced by probiotic fermentation, can enter host cells and function as histone deacetylase inhibitors, thereby altering gene expression patterns (usually promoting anti-inflammatory genes and mucin genes) [184]. SCFAs also bind G-protein-coupled receptors on enteroendocrine cells, stimulating hormone release (like PYY or GLP-2) that influences gut barrier integrity and immune modulation [185]. A concrete example of probiotic molecular action comes from aquaculture: in fish, *Bacillus* probiotics were found to activate innate immune signaling via TLR pathways, leading to increased cytokine production (e.g., more IL-1β, IL-8 from macrophages), which in turn primed the fish’s adaptive immunity [46]. The probiotics fortified the mucosal immune barrier, evidenced by higher mucosal IgA levels and up regulation of genes related to antigen presentation and lymphocyte activation [186]. Consequently, fish could mount a stronger immune response upon pathogen exposure. Such studies often employ transcriptomic analysis (RNA sequencing) of fish gut or gill tissues, revealing broad changes: dozens of immune and stress-response genes are differentially expressed in probiotic-treated fish vs. controls. For example, in Atlantic salmon distal intestine, 10 weeks of diets containing *Pediococcus acidilactici* produced RNA-seq signatures with 33 DEGs (27 up/6 down; FOS–BC vs. FOS) and 220 DEGs (174 up/46 down; GOS–BC vs. FOS–BC; BH-adjusted *q* ≤ 0.1), enrichments including ‘immune response’ and ‘response to stress,’ and up-regulation of il17 family and NADPH-oxidase genes (e.g., *duox/duox2*, *noxo1a/b*, *gpx1b*). Likewise, in zebrafish intestine, continuous exposure to *Lactobacillus casei* BL23 yielded 369 DEGs (237 up/132 down) at 35 dpf, affecting tight-junction, ECM–receptor interaction, and PPAR pathways [187,188].

### 5.6. Key Pathogens and Efficacy in Each Species

In cattle, important targets include *E. coli* (both enterotoxigenic strains in calves and Shiga-toxin producing O157 in feeder cattle), *Salmonella*, and *C. perfringens* (causative of calf enterotoxemia). Probiotics (like *L. acidophilus* NP51) have been shown to cut *E. coli* O157:H7 shedding by more than half in feedlot cattle [189]. As noted, Zhao et al. achieved near elimination of O157 in probiotic-treated calves. For *Salmonella*, some dairy calf studies saw reduced fecal *Salmonella* counts with *L. casei* supplementation [83]. However, results can vary by farm hygiene and Salmonella challenge load. In swine, neonatal and weaned piglets often face Enterotoxigenic *E. coli* (ETEC)—probiotics like *Lactobacillus reuteri* and *B. subtilis* have reduced ETEC attachment to the gut lining and lower edscours. One study found a multi-strain probiotic led to significantly fewer piglets shedding ETEC *F18* in feces (only 20% shed vs. 60% in controls) [190]. Swine probiotics also target *Salmonella* spp.: in weaned pigs challenged with *S. typhimurium*, a defined lactic-acid–bacteria mixture that included *Pediococcus pentosaceus* DPC6006 significantly reduced fecal *Salmonella* counts and disease severity; moreover, *Pediococcus acidilactici* supplementation reduced bacterial translocation to mesenteric lymph nodes after ETEC challenge [191]. Additionally, *Lawsonia* (ileitis) and *Brachyspira* (swine dysentery) are pathogens of interest—some evidence suggests certain *Faecalibacterium* or *Prevotella* probiotics might inhibit *Brachyspira* through butyrate production, though this is still being researched [192]. In poultry, as discussed, the biggest wins have been against *Salmonella*. Competitive exclusion products given to chicks can result in 99% lower *Salmonella* colonization if applied early enough. With *Campylobacter*, a combination of prebiotics and probiotics (synbiotics) sometimes yields ~1–2log CFU reductions in cecal load—not enough to eliminate the risk, but a useful decrease [193]. In broilers challenged with *Clostridium perfringens* (necrotic enteritis), a spore-forming *Bacillus* probiotic significantly suppressed disease severity: small-intestine lesion score fell from 2.17 to 1.13 (−48%; *p* < 0.001) and duodenal lesions from 1.13 to 0.38 (−66%; *p* < 0.001) at 2 days post-infection, with overall feed conversion numerically improved across d1–42 (FCR 1.65 vs. 1.67; *p* = 0.101) [194]. In aquaculture, probiotics have been successful against Vibrio species (like *V. harveyi*, *V. parahaemolyticus* causing AHPND in shrimp). By seeding shrimp tanks with beneficial *Bacilli*, farmers observed a dramatic drop in *Vibrio* counts in water and shrimp guts [46]. *Aeromonas hydrophila* is a ubiquitous freshwater pathogen that causes ulcerative disease in many cultured fish; *Lactobacillus* probiotics can reduce mortality by enhancing innate immunity—for example, feeding *L. plantarum* VSG3 (10^8 CFU g^−1^) to rohu for 60 days significantly increased serum lysozyme and phagocytic activity and yielded 77.7% post-challenge survival at 10 days after *A. hydrophila* infection (*p* < 0.05). Probiotics may also reduce fecal shedding of Salmonella in dogs on raw diets (though this has not been deeply studied yet). In cats, probiotics might help control *T. foetus* (a protozoal cause of colitis) indirectly by boosting native flora that outcompete the protozoa—a hypothesis under investigation. Probiotic efficacy is often quantified by pathogen load reductions or disease incidence reductions. By four weeks, almost 50% of probiotic birds had cleared Salmonella entirely (0 CFU in ceca) compared to only ~10% of controls [141]. In cattle, a direct-fed *Lactobacillus* trial showed a 50% lower odds of *E. coli* O157 prevalence in probiotic-supplemented steers (8% vs. 15% shedding). Shrimp survival improvements provide an indirect quantification: an 86% survival in probiotic-treated, *Vibrio*-challenged fish vs. 50% in non-treated in one case [195]. These numbers, while variable, illustrate meaningful pathogen control. In vivo studies commonly report ~1–3 log_10_ CFU reductions in probiotic/competitive-exclusion groups (e.g., 1–2 log_10_ decreases in broiler cecal/colon *Campylobacter* at slaughter age; 1.5–4 log_10_ decreases with selected probiotic isolates). Moreover, risk modeling indicates that 2–3 log_10_ reductions in broiler cecal *Campylobacter* translate into ~42–58% lower public-health risk [196,197].

**Table 2:** Environmental and sustainability aspects of probiotics in animal production.

Aspect	Mechanism	Outcome	References
Environmental sustainability of probiotics	Reduce pollution, mitigate greenhouse gases, enhance resource efficiency, combat AMR	Probiotics contribute to sustainable animal agriculture	[44,170,171]
Reducing methane emissions in livestock	Alter rumen microbial populations; suppress methanogenic archaea; redirect H_2_ to VFAs	15–20% less methane in dairy cows; 5% increase in milk; improved feed efficiency	[113,173,174,175,176]
Improving water quality in aquaculture	Probiotics (*B. subtilis*, *L. plantarum*) suppress pathogens; enhance nutrient cycling; degrade organic matter; remove NH_3_ and NO_2_^−^	20% reduction in shrimp mortality; 30% improved growth; fewer chemical treatments; healthier water	[176,177,178,179,180]
Reducing antibiotic usage	Competitive exclusion, bacteriocin production, immune modulation	Reduced disease incidence; 30% less post-weaning diarrhea in swine; improved growth performance; lower AMR spread	[184,185,186,187,188,189]
Sustainable farming practices	Optimize nutrient utilization; reduce nutrient excretion; improve gut absorption; use probiotic-treated manure	10% better FCR in poultry; 20% less N and P excretion in swine; enhanced soil health; improved animal welfare	[190,191]
Molecular mechanisms of probiotic action	Ligand interactions with TLRs/NODs; activate MAPK/NF-κB pathways; SCFA production; histone modification; GPCR signaling	Up-regulation of immune, barrier, and anti-inflammatory genes; stronger gut/immune barrier; improved host resilience	[192,193,194,195,196]
Key pathogens and efficacy in each species—Cattle	Target *E. coli* O157, *Salmonella*, *C. perfringens*; *Lactobacillus NP51*, *L. casei*	50% reduction in E. coli O157 shedding; reduced Salmonella counts	[72]
Key pathogens and efficacy in each species—Swine	Target ETEC, *Salmonella*, *Lawsonia*, *Brachyspira*; *Lactobacillus reuteri*, *B. subtilis*, *Pediococcus*	Reduced ETEC shedding (20% vs. 60% controls); reduced Salmonella translocation	[43,192]
Key pathogens and efficacy in each species—Poultry	Target *Salmonella*, *Campylobacter*, *C. perfringens*; *Bacillus*-based DFMs	99% lower Salmonella colonization; 1–2 log_10_ reduction Campylobacter; 48–66% lower NE lesions	[6,99]
Key pathogens and efficacy in each species—Aquaculture	Target *Vibrio* spp., *Aeromonas hydrophila*; *Bacillus* spp., *L. plantarum*	86% survival in Vibrio-challenged shrimp; 77.7% survival in fish; reduced pathogen load	[46,47,48]

## 6. Conclusions

Probiotic supplementation has been shown to exert multifaceted effects on animal physiology, contributing to improved health status, enhanced growth performance, and increased sustainability across multiple species. Their mechanisms of action include modulation of the gastrointestinal microbiota, regulation of host immune responses, and mitigation of pathogenic colonization, thereby reducing the necessity for antibiotic interventions and supporting evidence-based advancements in animal husbandry and veterinary medicine. In livestock, probiotics contribute to improved feed efficiency, reduced methane emissions, and enhanced growth performance, aligning with global goals for sustainable farming. For companion animals, probiotics play a critical role in managing gastrointestinal health, alleviating stress through the gut–brain axis, and addressing conditions such as allergies and behavioral disorders.

Despite these advantages, significant challenges remain. Variability in probiotic efficacy across species, strains, and environmental conditions continues to limit their predictable outcomes. Regulatory frameworks for probiotics in animal feed lack global standardization, creating barriers to commercialization and widespread adoption. Additionally, the cost of probiotics, particularly for small-scale farmers, hinders their accessibility and practical implementation. Looking ahead, the future of probiotics lies in precision and innovation. Advances in microbiome research and sequencing technologies offer the opportunity to develop targeted probiotic strains tailored to specific species, individual microbiota profiles, or production systems. There is also growing interest in genetically engineered probiotics and in the use of probiotics’ post-biotic products for targeted health outcomes. Addressing current challenges, such as strain efficacy variability and regulatory approval processes, through scientific and technological innovation will be crucial for wider adoption. Integration of probiotics with host genomics and metabolomics will further enhance their effectiveness and enable personalized animal health solutions. Expanding applications of probiotics into underexplored areas, such as wildlife conservation and exotic pet care, could open new frontiers in animal health. Moreover, the role of probiotics in climate change mitigation, through reduced methane emissions and improved resource efficiency, underscores their importance in addressing global environmental challenges. Moreover, the use of probiotics aligns with efforts to combat antimicrobial resistance (AMR), which poses significant risks to both human and animal health. By serving as alternatives to prophylactic antibiotics, especially in intensive farming systems where disease pressure is high, probiotics can reduce the selection for antibiotic-resistant pathogens. This benefit underscores the public health importance of integrating probiotics into animal management.

To fully unlock the potential of probiotics, a multidisciplinary approach is essential. Collaboration among researchers, industry stakeholders, policymakers, and farmers will drive innovation, streamline regulatory processes, and increase awareness about the benefits of probiotics. With continued research and development, probiotics have the potential to revolutionize animal health and farming practices, creating a more sustainable and ethical future for animal agriculture.

## Data Availability

The original contributions presented in this study are included in the article. Further inquiries can be directed to the corresponding author(s).
